# Individualized repetitive intravenous immunoglobulin therapy for a 79-year-old patient with GlyR antibody-positive PERM: a case report and literature review

**DOI:** 10.3389/fimmu.2026.1867563

**Published:** 2026-07-07

**Authors:** Xiaojun Liu, Jianwei Zhu, Yuanhong Lei, Xinyuan Yu, Xiujuan Mi

**Affiliations:** Department of Encephalopathy, Chongqing Traditional Chinese Medicine Hospital (The First Affiliated Hospital of Chongqing University of Chinese Medicine), Chongqing, China

**Keywords:** case report, glycine receptor antibody, intravenous immunoglobulin, progressive encephalomyelitis with rigidity and myoclonus, stiff-person syndrome

## Abstract

Progressive encephalomyelitis with rigidity and myoclonus (PERM) is a rare, life-threatening variant of stiff-person spectrum disorder that is primarily associated with anti-glycine receptor (GlyR) antibodies. Although advanced age and intensive care unit admission have been associated with poor outcomes for patients with GlyR antibody-positive PERM, detailed management strategies for elderly patients with multiple comorbidities remain limited. We report the successful individualized management of a 79-year-old male with glycine receptor 1 (GlyR1) antibody-positive PERM, who is among the oldest reported survivors of severe PERM. The patient initially presented with paroxysmal limb spasms, which were misattributed to spinal degeneration and a minor thalamic infarction. The subsequent development of a prominent startle response, respiratory failure, and autonomic instability prompted an autoimmune evaluation, confirming the presence of anti-GlyR1 antibodies in both serum and cerebrospinal fluid. High-dose corticosteroid pulse therapy was avoided because of an active pulmonary infection and comorbidity-related safety concerns. Intravenous immunoglobulin (IVIg) was selected as the initial immunomodulatory therapy, while plasma exchange was deferred and later discontinued after a single session because of procedure-related complications and hemodynamic instability. The patient received three sequential courses of IVIg; in addition, a single 20-mg subcutaneous dose of ofatumumab was administered as individualized off-label sequential/salvage immunotherapy. A major clinical improvement, including a reduction in spasms, successful extubation, antibody seroconversion, and excellent functional recovery with a modified Rankin scale (mRS) score of 1, occurred after repeated IVIg courses. At the 12-month follow-up, the patient remained clinically stable and had sustained negative GlyR1 antibody results. This case report highlights the importance of the early recognition of stimulus-sensitive spasms and the startle response in elderly patients with misleading age-related imaging findings. These findings also suggest that repeated IVIg courses may represent a feasible salvage strategy when conventional immunotherapies are limited, but the independent therapeutic contribution of ofatumumab remains uncertain. Serial GlyR1 antibody titers may be useful for monitoring the treatment response, although this result requires confirmation in larger studies.

## Introduction

Stiff-person syndrome (SPS) is a rare autoimmune neurological disorder characterized by fluctuating muscle rigidity and painful, stimulus-sensitive spasms (1, 2). It is most commonly associated with antibodies against glutamic acid decarboxylase 65 (GAD65), which impair γ-aminobutyric acid (GABA)-mediated inhibitory neurotransmission (2). Progressive encephalomyelitis with rigidity and myoclonus (PERM) represents the rarest and most severe subtype of stiff-person spectrum disorder. In addition to rigidity and spasms, PERM is distinguished by brainstem involvement, long-tract signs, autonomic dysfunction, myoclonus, and a prominent startle response (3, 4). Unlike classical SPS, PERM is more frequently associated with antibodies against the glycine receptor (GlyR), which target GlyR-enriched inhibitory synapses in the brainstem and spinal cord and thereby contribute to central nervous system hyperexcitability through impaired glycinergic inhibition (2, 5, 6).

Although PERM can affect individuals across different age groups, its diagnosis and management in elderly patients present particular challenges. Early manifestations may be subtle, focal, or atypical and may be misattributed to more common age-related disorders such as cerebrovascular disease or degenerative spinal disease. In addition, elderly patients often have multiple comorbidities, reduced physiological reserves, and a lower tolerance to conventional immunotherapies, which may increase the risk of treatment-related complications (2). A recent study of 41 patients with GlyR antibody-positive PERM reported a median age at onset of 58 years, a male predominance of 88%, and an intensive care unit admission rate of 51%. Importantly, advanced age and intensive care unit admission were identified as independent predictors of poor outcomes; the median age was 70 years among deceased patients compared with 54 years among survivors (7). Despite these observations, data on individualized therapeutic strategies for elderly patients with multiple comorbidities and severe PERM remain limited, and the reported outcomes in this subgroup are often unfavorable (8–10).

Here, we report a 79-year-old male with GlyR1 antibody-positive PERM who initially presented with focal limb spasms that were misattributed to spinal or vascular etiologies. The subsequent development of a prominent startle response, respiratory failure, and autonomic instability led to autoimmune testing and confirmed the diagnosis. Because of an active pulmonary infection and comorbidity-related safety concerns, high-dose corticosteroid pulse therapy was avoided, and the treatment approach was individualized accordingly. This case report illustrates the diagnostic challenges and therapeutic considerations in managing severe PERM in an elderly patient with multiple comorbidities.

## Case presentation

A 79-year-old male with an 8-day history of paroxysmal painful spasms of the left lower limb was admitted to our hospital. During each episode, he experienced tightness and pain involving the skin and muscles below the left knee, resulting in difficulty standing and walking. The episodes occurred approximately ten times per day, without identifiable triggers. He had no limb numbness or persistent weakness, no dizziness or headache, no nausea or vomiting, no vertigo, no upward eye deviation, no facial twitching, and no urinary or fecal disturbance. He had no personal or family history of psychiatric or autoimmune disorders.

The patient had a medical history of type 2 diabetes mellitus, chronic obstructive pulmonary disease, and osteoporosis. Before admission, he had been evaluated at two other hospitals. Lumbar spine imaging showed degenerative changes and osteoporosis, whereas brain computed tomography revealed lacunar infarcts in the bilateral basal ganglia and periventricular regions. A spinal or vascular etiology was initially suspected, but no targeted treatment was administered.

A neurological examination at admission showed that the patient was alert and cooperative, with clear speech and intact orientation, comprehension, naming, repetition, reading, writing, memory, abstract thinking, and executive function, although calculation was mildly impaired. The cranial nerve examination was unremarkable: the pupils were equal and reactive to light, ocular movements were full, and ptosis, diplopia, facial weakness, bulbar dysfunction, dysphagia, choking, or dysarthria was not present. The motor examination revealed increased muscle tone in the left lower limb, with Medical Research Council grade 5 strength proximally and grade 5− strength distally; muscle strength and tone were otherwise normal in the remaining limbs. The sensory examination showed reduced pinprick, temperature, and vibration sensation below the left thigh, whereas sensation in the other limbs was essentially normal. Tendon reflexes were symmetrical and graded as 2+ bilaterally, with no patellar or ankle clonus. Babinski, Chaddock, Oppenheim, and Gordon signs were negative bilaterally. Meningeal signs were absent. The autonomic examination at admission was unremarkable, with a normal skin color, temperature, and moisture; urinary or fecal incontinence was absent; and the dermographism test was negative. Paroxysmal spasms and jerking of the left lower limb were observed during the examination.

Brain diffusion-weighted imaging showed a punctate area of restricted diffusion in the right thalamus, initially suggestive of an acute lacunar infarct (**Figure 1A**). Magnetic resonance angiography showed no evidence of significant intracranial vascular stenosis (**Figure 1B**). The patient was initially treated with antiplatelet therapy, antispasmodic agents, including clonazepam and tizanidine, and neurotrophic support. However, his symptoms did not improve and progressively worsened.

**Figure 1:**
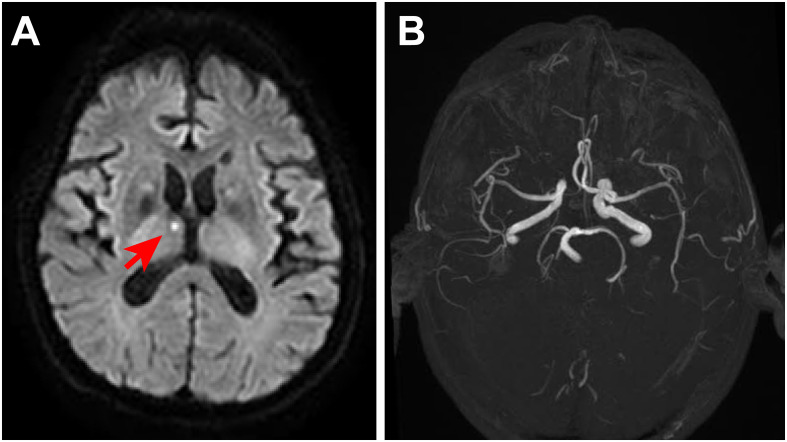
Brain magnetic resonance imaging (MRI) and magnetic resonance angiography (MRA) findings at admission. **(A)** Diffusion-weighted imaging (DWI) reveals a punctate area of restricted diffusion in the right thalamus (red arrow), which was initially suggestive of an acute lacunar infarct. **(B)** Magnetic resonance angiography (MRA) demonstrating no evidence of significant intracranial vascular stenosis.

On Day 7 after admission, the patient developed bilateral lower-lobe pneumonia, and his limb spasms continued to worsen. On Day 10 after admission, he experienced a transient loss of consciousness during a meal, accompanied by an upward deviation of the eyes, limb rigidity, and profuse sweating, followed by respiratory failure. Emergency intubation was performed, and the patient was transferred to the neurological intensive care unit.

At this stage, a pronounced startle response became evident: minimal auditory stimuli or light touch could trigger generalized rigidity. EEG showed no epileptiform discharges, whereas EMG captured myoclonic activity. Given the high clinical suspicion of an autoimmune neurological disorder, lumbar puncture and autoimmune antibody testing were performed. A cell-based assay revealed anti-GlyR1 antibody positivity in both serum and cerebrospinal fluid, with a titer of 1:30 in each test (**Figure 2**). Tests for other autoimmune encephalitis-related antibodies, including anti-N-methyl-D-aspartate receptor (NMDAR), anti-leucine-rich glioma-inactivated 1 (LGI1), anti-contactin-associated protein-like 2 (CASPR2), anti-γ-aminobutyric acid type A receptor (GABA_A receptor), anti-γ-aminobutyric acid type B receptor (GABA_B receptor), anti-α-amino-3-hydroxy-5-methyl-4-isoxazolepropionic acid receptor (AMPAR), and anti-glutamic acid decarboxylase 65 (GAD65) antibodies, were negative. A paraneoplastic antibody panel, including anti-Hu, anti-Yo, anti-Ri, anti-Ma2, and anti-amphiphysin antibodies, was also negative. Based on the clinical phenotype and results of the antibody tests, a diagnosis of GlyR1 antibody-positive progressive encephalomyelitis with rigidity and myoclonus was established.

**Figure 2:**
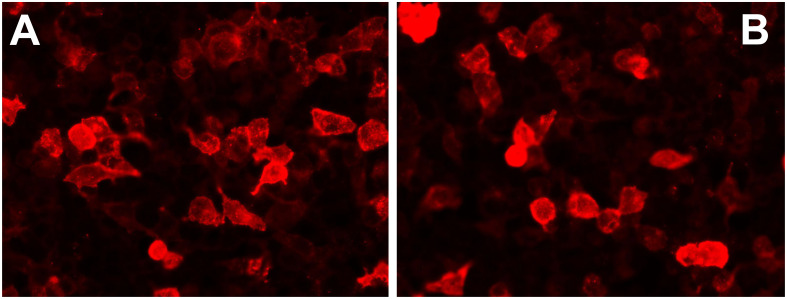
Detection of anti-glycine receptor 1 (GlyR1) antibodies using a cell-based assay (CBA). Indirect immunofluorescence staining of HEK293 cells transfected with the GlyR1 subunits revealed positive IgG staining (red) in both **(A)** serum and **(B)** cerebrospinal fluid (CSF), each with a titer of 1:30.

Considering the patient’s active pulmonary infection, poor glycemic control with a glycated hemoglobin (HbA1c) level of 9.5%, osteoporosis, and potential gastrointestinal bleeding risk, high-dose corticosteroid pulse therapy was avoided. Intravenous immunoglobulin (IVIg, 0.4 g/kg/day for 5 consecutive days) was selected as the initial immunomodulatory therapy and was started on Day 10 after admission. Plasma exchange was deferred at this stage because IVIg was considered the safer and more feasible initial option, but it was not excluded from later consideration.

After the first course of IVIg, the patient’s infection-related parameters improved, and ventilatory support was temporarily reduced on Day 17 after admission. However, on Day 20 after admission, respiratory distress recurred, and his disease severity, as quantified by the Clinical Assessment Scale in Encephalitis (CASE), reached a peak score of 15 on a 0–27 scale, with higher scores indicating greater severity. A repeat chest CT showed no significant worsening of pneumonia. After a reassessment of ongoing disease activity and treatment limitations, anti-CD20 therapy with ofatumumab was administered as an individualized off-label sequential/salvage immunotherapy with a single 20 mg subcutaneous dose. No additional loading or maintenance doses of ofatumumab were administered because no clear clinical improvement was observed after the administration of the single dose, and continued B-cell-depleting therapy was considered unsuitable given the patient’s advanced age, recent pulmonary infection, and overall safety concerns.

During the subsequent clinical course, the patient developed psychiatric manifestations, paroxysmal tachycardia and bradycardia, and fluctuating blood pressure. On Day 33 after admission, because the patient remained clinically unstable and repeated anti-GlyR1 antibody testing showed persistent positivity in both serum and cerebrospinal fluid, with a titer of 1:30 in each, plasma exchange was attempted on the same day. However, it was discontinued after a single session, with an exchange volume of 1200 mL calculated according to the patient’s low body weight of approximately 40 kg because of puncture-site hematoma at the right femoral vein access site and hemodynamic instability.

A second course of IVIg was administered on Day 35 after admission to address the persistently positive antibody titers and ongoing neurological symptoms. After the second IVIg course, the frequency of myoclonic jerks decreased significantly, and the CASE score decreased to 6. Because residual symptoms persisted, a third course of IVIg was administered on Day 63 after admission. After this course, the patient’s autonomic symptoms and psychiatric manifestations gradually stabilized, and the CASE score decreased to 3.

On Day 73 after admission, the patient was successfully extubated and transferred to the general ward for rehabilitation, with a CASE score of 2. Repeat anti-GlyR1 antibody testing revealed seroconversion to a negative result in both serum and cerebrospinal fluid. The patient was discharged on Day 80 after admission, with a CASE score of 1.

At the 1-month follow-up, the patient was able to walk independently with no recurrence of limb jerking, and anti-GlyR1 antibody testing results remained negative. At the 12-month follow-up, he remained clinically stable, with a CASE score of 0, a modified Rankin scale score of 1, and sustained negative anti-GlyR1 antibody results. The full clinical course and treatment timeline are illustrated in **Figure 3**.

**Figure 3:**
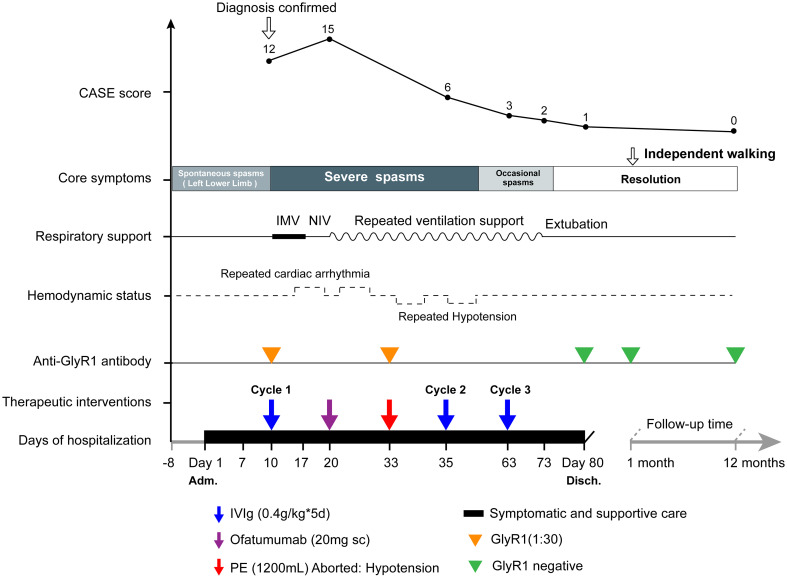
Clinical timeline summarizing disease progression, therapeutic interventions, and outcomes. The graph illustrates dynamic changes in the Clinical Assessment Scale in Encephalitis (CASE) score, core neurological symptoms, respiratory support requirements, hemodynamic status, and anti-GlyR1 antibody status during hospitalization from Day 1 to Day 80 after admission and during the subsequent 12-month follow-up. The patient received continuous symptomatic and supportive care from admission to discharge. The blue arrows indicate the courses of intravenous immunoglobulin (IVIg, 0.4 g/kg/day for 5 consecutive days). The purple arrow indicates the administration of a single 20-mg subcutaneous dose of ofatumumab on Day 20 after admission as individualized off-label sequential/salvage immunotherapy. The red arrow indicates a single plasma exchange session on Day 33 after admission, with an exchange volume of 1200 mL, which was aborted because of hypotension/hemodynamic instability and puncture-site hematoma. The patient was successfully extubated on Day 73 after admission and discharged on Day 80 after admission. Anti-GlyR1 antibody testing results are marked by triangles: orange triangles indicate positive results with a titer of 1:30, and green triangles indicate negative results. Abbreviations: CASE, Clinical Assessment Scale in Encephalitis; GlyR1, glycine receptor 1; IVIg, intravenous immunoglobulin; PE, plasma exchange; IMV, invasive mechanical ventilation; NIV, noninvasive ventilation; sc, subcutaneous; Adm., admission; Disch., discharge.

## Discussion

This case report documents the successful individualized management of severe GlyR1 antibody-positive PERM in a 79-year-old male with multiple comorbidities. As summarized in **Table 1**, elderly patients with anti-GlyR antibody-positive PERM generally have poor outcomes, particularly when respiratory failure, autonomic instability, infection, or limited tolerance to immunotherapy coexist. The present patient was among the oldest reported survivors of severe PERM and achieved independent ambulation, sustained antibody seroconversion, and a modified Rankin scale score of 1 at the 12-month follow-up. The clinical value of this case report lies not only in the favorable outcome but also in the therapeutic decision-making process under conditions in which conventional high-dose corticosteroid therapy and plasma exchange were limited by the infection risk, comorbidity burden, and procedure-related complications.

**Table 1:** Clinical features and outcomes of GlyR antibody-positive PERM patients aged ≥ 60 years.

Case/reference	Age/sex	Clinical presentation	Antibody type	Comorbidities	Immunotherapy regimen	Outcome
Present case	79/M	Limb spasms, prominent startle response, respiratory failure, autonomic instability	GlyR1	T2DM, COPD, pneumonia, osteoporosis	IVIg×3, ofatumumab	Independent ambulation, antibody seroconversion, mRS score of 1
Yabata, 2025 (21)	80/M	Oculomotor dysfunction, autonomic failure	GlyR	—	—	Died
Winklehner, 2024 (10)	75/M	Myoclonus, pain, startle response, hallucinations, autonomic dysfunction	GlyR, GAD65	—	—	Died
Yuan, 2022 (8)	75/M	Spasms, PSH, respiratory failure	GlyR	VZV infection	IVMP, IVIg	Died after discharge against advice
Song, 2024 (9)	71/M	Orthostatic hypotension, diplopia, spasms	GlyR, GAD, GM1, GQ1b	Pulmonary embolism	IVMP, IVIg, PLEX, RTX, CYC	Died from pulmonary embolism and cardiac arrest
Irie, 2022 (22)	71/F	Unilateral facial palsy, rigidity, spasms	GlyR	SIADH	IVMP, IVIg, PLEX	Required walking aid
Qin, 2025 (Case 1) (23)	65/M	Spasms, respiratory failure	GlyR	—	IVIg, IVMP, MMF	Significant improvement

This table presents a nonexhaustive summary of GlyR antibody-positive PERM cases diagnosed in patients aged ≥ 60 years in studies identified through a PubMed search from January 2018 to April 2026. Cases are listed in descending order of age to highlight the clinical challenges in the geriatric population. The clinical outcomes and details of immunotherapy reflect the original descriptions provided by the respective authors. The mRS score for the present case was assigned as 1 based on independent ambulation and self-care at follow-up. IVIg, intravenous immunoglobulin; PLEX, plasma exchange; PSH, paroxysmal sympathetic hyperactivity; T2DM, type 2 diabetes mellitus; COPD, chronic obstructive pulmonary disease; VZV, varicella-zoster virus; SIADH, syndrome of inappropriate antidiuretic hormone secretion; RTX, rituximab; CYC, cyclophosphamide; IVMP, intravenous methylprednisolone; MMF, mycophenolate mofetil

The initial diagnostic challenge in this case reflects an important pitfall in elderly patients with PERM. The patient first presented with focal painful spasms of the left lower limb, accompanied by age-related imaging abnormalities, including lumbar degenerative changes and a small thalamic lacunar infarct. These findings initially biased the diagnostic reasoning toward spinal or vascular etiologies. However, the subsequent emergence of a prominent startle response, stimulus-sensitive generalized rigidity, respiratory failure, and autonomic instability was grossly disproportionate to these structural findings and prompted an autoimmune evaluation. This case report highlights a practical clinical message: in elderly patients, common imaging abnormalities should not preclude the consideration of autoimmune neurological disorders when paroxysmal painful spasms, stimulus sensitivity, a startle response, or fluctuating rigidity are clinically prominent. Early antibody testing is particularly important when the phenotype evolves to include respiratory dysfunction, autonomic instability, or neuropsychiatric manifestations.

A major challenge in this case was recurrent respiratory failure requiring prolonged ventilatory support. Bilateral lower-lobe pneumonia was present during the acute deterioration and likely contributed to the initial respiratory failure. However, the subsequent clinical course suggested that respiratory dysfunction could not be explained solely by the pulmonary infection. Respiratory distress recurred despite improvements in infection-related parameters, and repeat chest imaging did not show a significant progression of pneumonia. In the context of severe stimulus-sensitive rigidity, myoclonus, and autonomic instability, PERM-related neurogenic respiratory involvement was therefore considered an important contributing factor. GlyR antibodies may impair inhibitory glycinergic neurotransmission in the brainstem and spinal cord, where glycinergic pathways participate in motor and respiratory control (11). Accordingly, brainstem involvement, respiratory muscle rigidity or spasms, impaired central respiratory regulation, and autonomic instability may all contribute to respiratory failure in patients with severe PERM.

Autonomic dysfunction was also prominent during the critical phase, manifesting as paroxysmal tachycardia and bradycardia, fluctuating blood pressure, and recurrent hypotensive episodes. Previous reports have described severe autonomic instability in patients with PERM, including paroxysmal sympathetic hyperactivity, orthostatic hypotension, and profound blood pressure fluctuations (8, 9, 11). In our patient, hypotension was clinically important because it increased the risk of plasma exchange and contributed to the discontinuation of the procedure after one session. Nevertheless, autonomic dysfunction in critically ill patients should be interpreted cautiously. Sedative exposure, infection-related circulatory instability, hypovolemia, hypoalbuminemia, and hypercapnia may also contribute to hypotension or respiratory deterioration. Therefore, the respiratory and circulatory abnormalities in this case were most likely multifactorial, with both pulmonary infection and PERM-related neurogenic mechanisms contributing to the clinical course.

With respect to immunotherapy, the patient’s condition presented a major therapeutic dilemma. High-dose corticosteroids, IVIg, and plasma exchange are commonly used to treat PERM (12, 13). In this case, however, high-dose corticosteroid pulse therapy was avoided because of an active pulmonary infection and comorbidity-related safety concerns, including poor glycemic control with an HbA1c level of 9.5%, osteoporosis, and a potential gastrointestinal bleeding risk. IVIg was therefore selected as the initial immunomodulatory therapy. Plasma exchange was deferred initially rather than excluded, but when it was later attempted on Day 33 after admission, it had to be discontinued after a single session because of puncture-site hematoma and hemodynamic instability. This sequence highlights that treatment selection in elderly patients with severe PERM must be individualized according to both disease severity and treatment tolerance.

The patient received three courses of IVIg, and the temporal relationship between repeated IVIg administration and the clinical improvement was notable. After the first IVIg course, infection-related parameters improved, and ventilatory support was temporarily reduced. However, respiratory distress and severe neurological symptoms recurred, and anti-GlyR1 antibody titers remained positive on Day 33 after admission. After the second and third IVIg courses, the frequency of myoclonic jerks decreased, the patient’s autonomic and psychiatric manifestations stabilized, the patient was successfully extubated, and the anti-GlyR1 antibodies became negative. This temporal sequence suggests that repeated IVIg administration likely played a dominant therapeutic role in this case.

Ofatumumab was administered once as individualized off-label sequential/salvage immunotherapy because of the severity of the antibody-mediated disease and the limited suitability of conventional immunotherapies. A biological rationale for the use of anti-CD20 therapy in antibody-mediated neurological disorders has been established, and published experience with B-cell-depleting therapy in selected severe or refractory PERM cases has mainly involved rituximab (14–16). However, the selection of ofatumumab in this case was based on patient-specific feasibility and pharmacological considerations rather than stronger PERM-specific evidence. Rituximab remained a reasonable anti-CD20 option, but it would have required prolonged intravenous infusion, infusion-rate titration, monitoring for infusion-related reactions, and additional intravenous fluid exposure. In the setting of a cautious single-dose exploratory anti-CD20 intervention, ofatumumab was considered more suitable for this specific patient because it was accessible, is a fully human anti-CD20 monoclonal antibody, and can be administered subcutaneously with a lower infusion burden. In addition, ocrelizumab was not readily available at our institution in a timely manner, whereas ublituximab was not locally available at the time of treatment. However, ofatumumab has not been validated as a treatment for PERM, and the patient did not receive the standard dosing regimen used to treat multiple sclerosis (17). No clear clinical improvement was observed after a single dose of ofatumumab, and anti-GlyR1 antibody titers remained positive on Day 33 after admission. A major clinical improvement occurred only after the patient was administered subsequent IVIg courses. Therefore, no conclusion can be drawn from this single case regarding the efficacy, optimal dose, dosing frequency, or safety of ofatumumab in treating PERM; its use should be interpreted as a confounding exploratory intervention rather than evidence of therapeutic efficacy. This selection should not be interpreted as evidence that ofatumumab is preferable to rituximab, ocrelizumab, ublituximab, or other anti-CD20 agents in PERM.

To place this case in the context of reported elderly PERM cases, we summarized published GlyR antibody-positive PERM cases involving patients aged ≥60 years in **Table 1**. Overall, the outcomes in this age group appear unfavorable. Among the reported elderly cases identified, several patients died, and others had a residual disability requiring walking assistance, whereas only a minority achieved substantial functional recovery. The present patient was 79 years old, had multiple comorbidities, including type 2 diabetes mellitus, chronic obstructive pulmonary disease, osteoporosis, and pneumonia, and had restricted tolerance to conventional immunotherapies. Despite these high-risk features, he achieved independent ambulation, sustained antibody seroconversion, and an mRS score of 1 at the 12-month follow-up. This contrast highlights the potential clinical value of individualized treatment strategies for elderly patients with severe PERM, particularly when standard immunotherapeutic options are limited by infection, the comorbidity burden, or treatment-related complications.

The patient achieved seroconversion of anti-GlyR1 antibodies from a titer of 1:30 to negative, which paralleled his clinical recovery. This improvement was also reflected by a continuous decrease in the CASE score, from a peak score of 15 during the most critical phase to 0 at the 12-month follow-up. In this case, the CASE score provided a more detailed assessment of acute neurological symptoms, whereas the mRS score reflected long-term functional recovery and independence. The temporal association between antibody seroconversion and clinical improvement suggests that serial anti-GlyR1 antibody testing may be useful for monitoring the treatment response in selected patients. However, given the single-case nature of this observation, the value of anti-GlyR1 antibody titers as a biomarker requires further validation in larger cohorts.

Although only anti-GlyR1 antibodies were detected in our patient, previous reports have described the coexistence of multiple autoantibodies in patients with PERM. For example, PERM cases with concomitant GlyR, GAD65, AChR, Titin, GM1, or GQ1b antibodies have been reported in the literature (9, 18). These findings suggest that broader antibody screening may be useful when clinically feasible, particularly in patients with atypical or severe presentations. In addition, PERM may occur as a paraneoplastic neurological syndrome, with thymoma being one of the most frequently reported associated tumors (12, 19, 20). Therefore, tumor screening and long-term surveillance remain important in PERM patients. In our patient, initial paraneoplastic antibody testing was negative, and follow-up imaging over 12 months showed no evidence of malignancy; nevertheless, continued surveillance is warranted.

This study has several limitations. First, as a single case report, the findings cannot be generalized to all patients with PERM, particularly given the rarity and clinical heterogeneity of anti-GlyR antibody-positive disease. Second, the patient received overlapping interventions, including repeated IVIg courses, a single dose of ofatumumab, anti-infective treatment, respiratory support, and intensive supportive care. Therefore, although the temporal sequence suggests that repeated IVIg treatment likely played a dominant therapeutic role, the clinical improvement cannot be attributed exclusively to any single intervention.

Third, the single-dose use of ofatumumab was off-label and exploratory. The efficacy, optimal dose, dosing frequency, and safety of ofatumumab for treating this disease have not been validated. Fourth, respiratory and circulatory abnormalities occurred in the context of both severe PERM and a critical illness; pulmonary infection, sedative exposure, hypovolemia, hypoalbuminemia, and hypercapnia may have contributed to respiratory deterioration or hypotension. Therefore, the relative contributions of neurogenic mechanisms and systemic factors cannot be definitively separated.

Fifth, although paraneoplastic antibody testing was negative and follow-up imaging over 12 months showed no evidence of malignancy, whole-body PET–CT was not performed during the acute phase because of transport risks and clinical instability. The possibility of delayed tumor detection cannot be completely excluded, and continued surveillance is warranted. Finally, antibody testing was performed using cell-based assays, and the functional channel-blocking activity of the anti-GlyR1 antibodies was not assessed. Longer-term follow-up is ongoing to monitor for relapse, late tumor development, and sustained functional recovery.

## Conclusions

This case report highlights the diagnostic and therapeutic challenges associated with severe GlyR1 antibody-positive PERM in elderly patients with multiple comorbidities. In older patients, focal painful spasms and common age-related imaging abnormalities may obscure the diagnosis; the emergence of stimulus-sensitive spasms, a prominent startle response, respiratory dysfunction, autonomic instability, or neuropsychiatric manifestations should prompt early autoimmune antibody testing.

When conventional immunotherapies are limited by infection, comorbidities, or procedure-related complications, individualized treatment strategies are needed. In this patient, repeated IVIg courses were temporally associated with clinical recovery, successful extubation, antibody seroconversion, and favorable long-term functional outcomes, suggesting that repeated IVIg treatment may represent a feasible salvage option in selected high-risk elderly patients. The single-dose use of ofatumumab was selected for patient-specific feasibility reasons, including its fully human anti-CD20 structure, subcutaneous route of administration, lower intravenous infusion burden, and timely local availability; however, this exploratory intervention should not be overinterpreted, and its independent therapeutic contribution remains uncertain. Further studies and longer-term follow-up are needed to clarify the optimal treatment strategies, risk of relapse, and role of serial anti-GlyR1 antibody monitoring in patients with PERM.

## Data Availability

The original contributions presented in the study are included in the article/supplementary material. Further inquiries can be directed to the corresponding author.
